# Anterior annular and left ventricular outflow tract enlargement for redo truncal valve replacement for truncus arteriosus

**DOI:** 10.1016/j.xjtc.2025.09.009

**Published:** 2025-09-19

**Authors:** Hironobu Nishiori, Masashi Kabasawa, Mitsuru Aoki, Takahiro Muramatsu, Hiroki Ikeuchi, Kozo Matsuo

**Affiliations:** a Department of Cardiovascular Surgery, Chiba Kaihin Municipal Hospital, Chiba, Japan; b Department of Cardiovascular Surgery, Chiba University Hospital, Chiba, Japan

**Figure:**
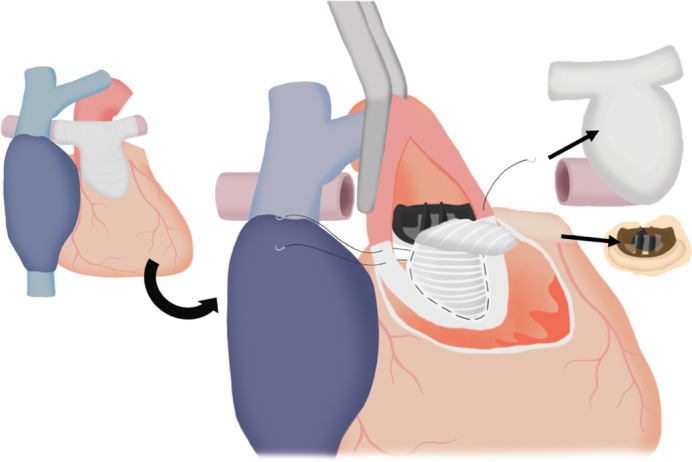
Anterior annular enlargement enabled retruncal valve replacement after TA repair.

**Central Message**

Anterior annular incision allows safe annular enlargement and enables retruncal valve replacement in patients with truncus valve dysfunction after previous truncus arteriosus repair.

Despite advances in truncus arteriosus (TA) repair, late truncal valve dysfunction remains challenging. Previous studies have demonstrated that 12% to 28% of primary truncus arteriosus repairs require a simultaneous truncal valve intervention, including truncal valve replacement.^1^, ^2^, ^3^, ^4^ In certain cases, a small truncal annulus or patient-prosthesis mismatch after truncal valve replacement necessitates annular enlargement to implant a larger prosthesis. We experienced a case of retruncal valve replacement using an anterior incision for annular enlargement in a patient with structural valve deterioration after truncal valve replacement, pulmonary artery (PA) reconstruction, and right ventricular outflow tract reconstruction (RVOTR) for TA. Limited literature exists on this approach, which we describe here. Review board approval was not required for this deidentified single case report. The patient provided written informed consent for the publication of this report, including clinical details and images.

## Case Description

The patient was a 22-year-old woman who, at 7 months of age, underwent ventricular septum defect (VSD) closure and PA reconstruction using the Réparation à l’Étage Ventriculaire method for TA. At the age of 7 years, she developed severe truncal valve insufficiency and bilateral PA stenosis, for which she underwent truncal valve replacement with a 19-mm On-X valve (Senko Medical Instruments Mfg Co, Ltd), bilateral PA plasty using bovine pericardium, and RVOTR with an expanded polytetrafluoroethylene (PTFE) monocuspid valve transannular patch.

**Figure 1:**
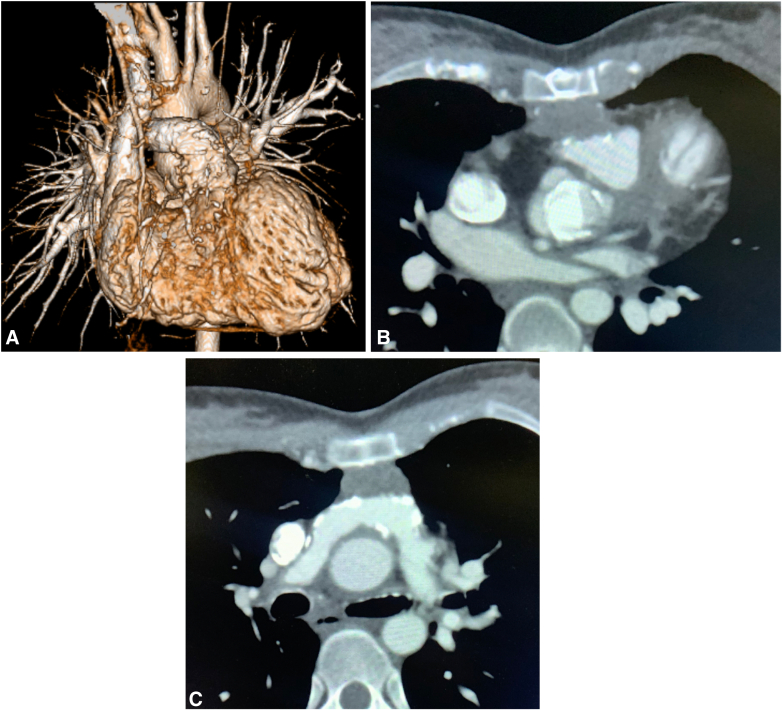
Three-dimensional computed tomography imaging (A), and axial view demonstrating compression of the right ventricular outflow tract by a depressed sternum (B) and severe calcification of the bilateral pulmonary arteries (C).

At 22 years old, she began complaining of exertional dyspnea. Computed tomography (CT) imaging demonstrated pannus formation around the On-X valve, as well as significant calcification of the transannular patch and both PAs. In addition, the sternum was depressed, compressing the right ventricular outflow tract from the anterior side (Figure 1, *A-C*). Cardiac catheterization revealed pressure gradients of 48 mm Hg in the right PA and 40 mm Hg in the left PA. The coronary anatomy consisted of a single coronary artery arising from the left coronary cusp that bifurcated into the right and left coronary arteries, with otherwise-normal distal courses. Transthoracic echocardiography showed a left ventricular ejection fraction of 61% and a peak velocity of 4.2 m/s across the On-X valve, and moderate pulmonary regurgitation.

**Figure 2:**
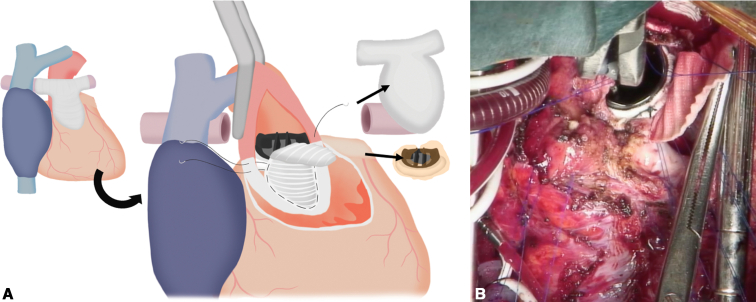
Intraoperative schema (A) and view (B) of annular enlargement and valve implantation: longitudinal anterior incision through the previous VSD patch with using a 24-mm Triplex graft for left ventricular outflow tract enlargement, and intraoperative view of implantation of a 21-mm On-X valve in the enlarged annulus. *VSD*, Ventricular septum defect.

**Figure 3:**
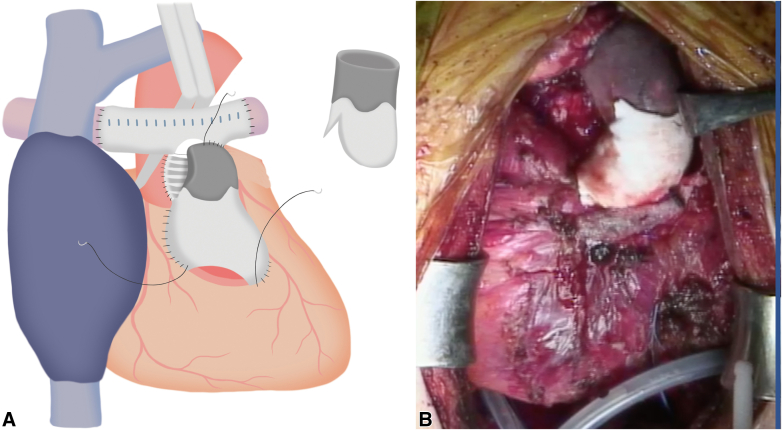
Intraoperative schema (A) and views (B) of pulmonary artery plasty and right ventricular outflow tract reconstruction. Bilateral pulmonary artery plasty using a 12-mm Gore-Tex graft, and right ventricular outflow tract reconstruction with a custom three-cusped bovine pericardium conduit reinforced with 0.6-mm ePTFE in the ventricular portion and 0.1-mm ePTFE valve cusps. *ePTFE*, Expanded polytetrafluoroethylene.

On the basis of these findings, the patient was diagnosed with structural valve deterioration, moderate pulmonary regurgitation, and bilateral PA stenosis. We decided to perform retruncal valve replacement with anterior annular enlargement, rebilateral PA plasty, and re-RVOTR. During the operation, the right femoral artery was exposed, and because its diameter was small, an artificial graft was preemptively anastomosed to ensure adequate flow during cardiopulmonary bypass. During pericardial dissection, bleeding from the left PA necessitated the establishment of cardiopulmonary bypass via the exposed right femoral artery and vein. Consistent with the CT findings, marked calcification extending from the right ventricular outflow tract to both PA was debrided, and both PA were transected at the peripheral margin of the previous bovine pericardium patch (Video 1). An incision was made in the anterior aspect of the aorta and was extended longitudinally, beyond the On-X valve to the center of the previous VSD patch, in the direction of a Konno incision. The 19-mm On-X valve and its associated pannus were excised. To achieve annular enlargement, a 24-mm Triplex graft (Vascutek Terumo Inc), trimmed into a boat shape with a 2.5-cm width, was sutured to the old VSD patch at the site of the longitudinal incision. A 21-mm On-X valve was then implanted, and the remaining patch was used to reconstruct the aortic wall (Figure 2, *A* and *B*). Subsequently, bilateral PA plasty was performed by anastomosing a 12-mm Gore-Tex graft (W.L. Gore & Associates), approximating the diameter of the native PA to both PAs. Finally, RVOTR was completed using a custom-made conduit. This conduit was a 25-mm, 3-cusped construct; it was fashioned from bovine pericardium, whereas the right ventricular portion was reinforced with a 0.6-mm ePTFE patch, and the valve cusps were created from 0.1-mm ePTFE patch (Figure 3, *A* and *B*).

The cardiopulmonary bypass time was 365 minutes, and aortic crossclamp time was 199 minutes. The postoperative course was uneventful, and after rehabilitation, the patient was discharged home on postoperative day 29. A CT performed 6 months postoperatively showed no stenosis from the right ventricular outflow tract to either PA. Six-month postoperative transthoracic echocardiography demonstrated normalization of On-X valve peak velocity to 2.0 m/s, and mild pulmonary regurgitation.

## Discussion

Although surgical outcomes for TA have improved over the years, there has been an increasing trend in late interventions on the truncal valve, with some cases ultimately requiring truncal valve replacement.^5^ As patients grow, patient-prosthesis mismatch or structural valve deterioration may necessitate retruncal valve replacement, during which annular enlargement is sometimes required to accommodate a larger prosthesis. As demonstrated in our case, performing an anterior longitudinal incision—like the Konno incision—via an approach from the anterior right ventricle can be advantageous in redo procedures, especially when concomitant RVOTR is required, because it minimizes the extent of dissection while providing effective annular enlargement. The previous VSD patch was Dacron. Because intraoperative inspection revealed no severe calcification or friability, we were able to extend the anterior longitudinal incision through the existing patch without complete replacement; otherwise, complete excision and replacement with a larger patch would have been required.

In contrast, in cases like our case, whose sternum was somewhat depressed, careful valve selection is required to balance the sizes of the truncal and pulmonary valves. Although anterior annular enlargement could allow implantation of a prosthesis 2 to 3 sizes larger in the aortic position, we opted for a 21-mm valve—one size larger—considering the need to preserve space for the pulmonary valve. For the pulmonary valve, there was insufficient space for a bioprosthetic valve; therefore, we used a custom-made 25-mm, 3-cusped conduit. Although an option exists for a bulging-sinus–type ePTFE conduit, we expected our custom-made conduit with deeper cusp coaptation would be less prone to pulmonary regurgitation even if slight deformation occurred beneath the sternum. We selected a bovine-pericardium conduit to preserve the option of future transcatheter pulmonary valve replacement; while bovine pericardium carries a potential risk of late calcification, the valve cusps were fashioned from 0.1-mm ePTFE, which is less prone to calcification. Decellularized equine pericardium, which may reduce the risk of late calcification, is available in Europe but is not readily obtainable in Japan and was therefore not used. Although homografts are also an effective option, their availability in Japan is extremely limited, making their use difficult.

There are reports that, after an arterial switch and PA reconstruction for transposition of the great arteries using the Lecompte maneuver, performing neoaortic valve replacement and RVOTR with a Konno incision is effective for annular enlargement.^5^ Our experience suggests that an anterior annular enlargement is also useful for re-truncal valve replacement.

## Conclusions

We report a case of retruncal valve replacement with annular enlargement via an anterior annular incision, combined with bilateral PA plasty and RVOTR, after intracardiac repair, truncal valve replacement, and RVOTR for TA. The anterior annular incision is a valuable technique for achieving annular enlargement in retruncal valve replacement after TA repair.

## Conflict of Interest Statement

The authors reported no conflicts of interest.

The *Journal* policy requires editors and reviewers to disclose conflicts of interest and to decline handling or reviewing manuscripts for which they may have a conflict of interest. The editors and reviewers of this article have no conflicts of interest.

## Supplementary Data

Video 1 Intraoperative video of anterior annular and left ventricular outflow tract enlargement, redo truncal valve replacement, and right ventricular outflow tract reconstruction. Video available at: https://www.jtcvs.org/article/S2666-2507(25)00374-8/fulltext.
