# Supplementary figures and images for: Anterior annular and left ventricular outflow tract enlargement for redo truncal valve replacement for truncus arteriosus
Source: JTCVS Tech. 2025 Sep 19;34:161–4. doi: 10.1016/j.xjtc.2025.09.009 (PMC12683047; doi:10.1016/j.xjtc.2025.09.009)

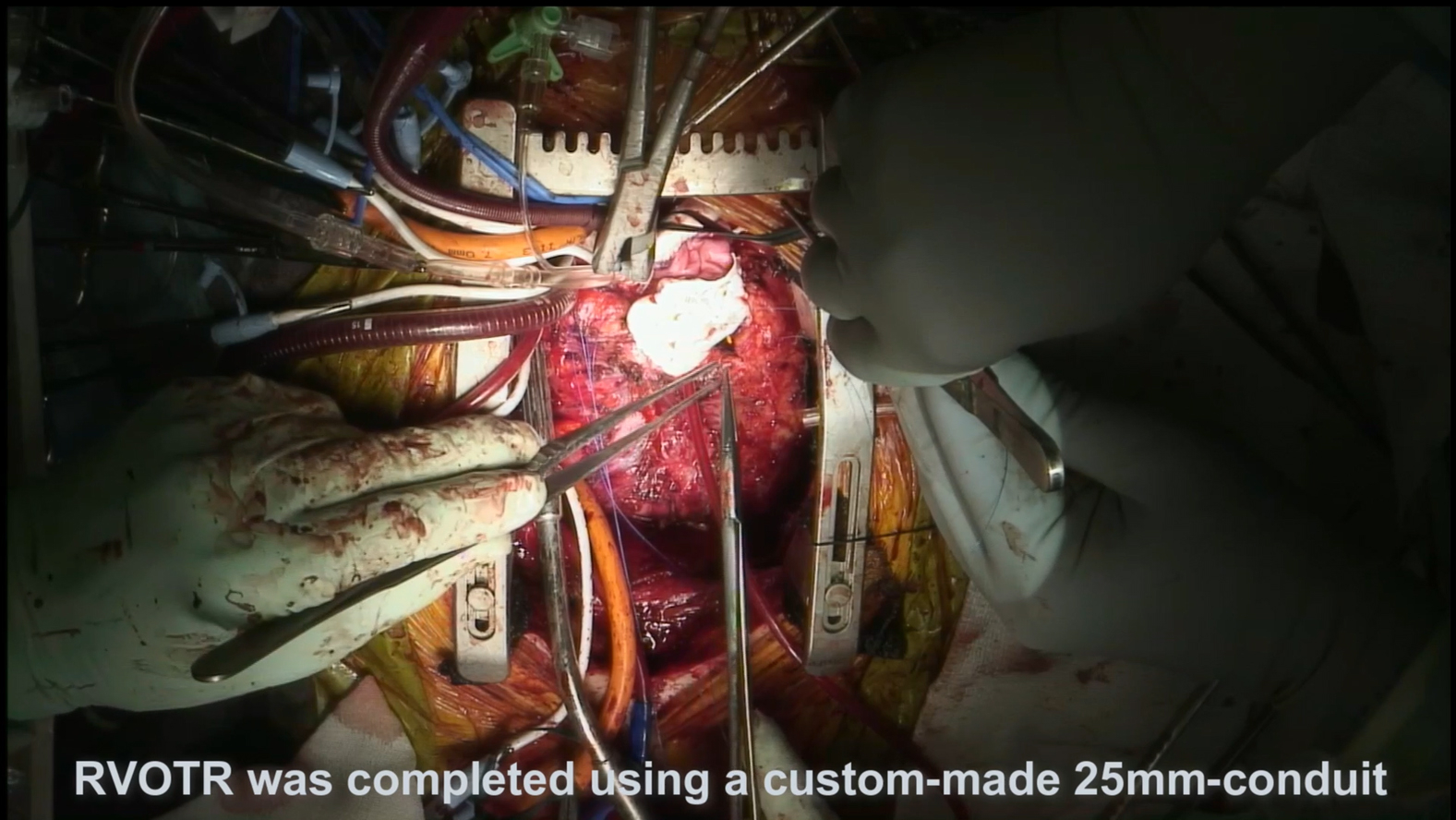

Supplement: Video 1 — Intraoperative video of anterior annular and left ventricular outflow tract enlargement, redo truncal valve replacement, and right ventricular outflow tract reconstruction. Video available at: https://www.jtcvs.org/article/S2666-2507(25)00374-8/fulltext. [file fx2.jpg]
